# Assistive technology: opportunities for societal inclusion of persons with disabilities and independence of the elderly

**DOI:** 10.1186/s42490-023-00072-8

**Published:** 2023-07-03

**Authors:** Aliaa Rehan Youssef, Ahmed Morsy

**Affiliations:** 1 Department of Biomedical and System Engineering, Faculty of Engineering, Cairo University, Giza, Egypt; 2 Department of Orthopedic Physical Therapy, Faculty of Physical Therapy, Cairo University, Giza, Egypt

## Abstract

Assistive technology (AT) development worldwide aims to enhance the quality of life for persons with disabilities and elderly, yet its development and commercialization may face challenges. This collection aims at obtaining a better understanding of the hurdles that various stakeholders may face in the successful development and commercialization of AT.

## Main text

Assistive technology (AT) has undergone significant evolution in recent decades. AT has evolved from simple mechanical devices to advanced tools that incorporate technologies such as wearables, machine learning, computer vision, and the internet of medical things (IoMT). One example of this transformation is the shift from traditional white canes used by visually impaired individuals to smart canes that utilize google maps, Global position system (GPS), or proximity sensors. The use of AT is essential in promoting social integration of people with disabilities (PWDs), increasing their independence, and ultimately improving their quality of life. Additionally, as the global aging population continues to increase rapidly, the elderly may also rely on AT to address problems and risks associated with aging frailty. However, to achieve its full potential, AT must receive a significant boost for adoption worldwide, considering economic and societal constraints, especially in low- and middle-income countries.

AT devices typically follow a standard technology development life cycle, which involves progressing from research to the development and validation of prototypes, followed by commercialization and adoption by target customers. The AT development life cycle, however, does not always follow this process smoothly or sequentially as intended. Thus, it is not surprising that different ATs showed various degrees of success in following the aforementioned progression. For instance, the adoption rate of smart canes remains low despite maturing into commercialization. This was partly attributed to negative user experiences associated with traditional cane use. Additionally, the high cost of the smart cane as perceived by individuals with impaired vision and their communities is a major factor that limited its wide scale use [1].

Similarly, the adoption of AT based on wearable sensors for fall prevention in the elderly has been challenged by concerns about their added value, cost, and ease of use, among other factors [2]. It is notable that although ATs for elderly fall prevention have been validated in controlled settings and in a few clinical trials, it is still uncertain whether they are clinically effective as standalone preventive measures for reducing fall rates [3]. Conversely, brain computer interface (BCI) devices appear to be stuck in the research and development phases of the cycle as there are several crucial factors that must be addressed before they can be broadly commercialized. These factors include signal quality, hardware performance, user experience and comfort, and the overall cost of the device. Other examples in this category include exoskeletons and visual prosthetics [4–7]. Although the first visual prosthetic device was approved by the FDA in 2013, these AT devices are still far from achieving commercial viability.

Successful examples of AT development, commercialization, and adoption are also available. Hearing aids and powered wheelchairs are two of these examples. In addition, companies like Apple, Google, and Microsoft made efforts to make their products accessible for PWDs on personal and mobile computing platforms. This accessibility has helped thousands of PWDs, albeit with various degrees. Moreover, the adoption of virtual reality and serious games in AT domain is gaining momentum worldwide, particularly after the paradigm shift brought about by the COVID-19 pandemic which necessitated searching for alternatives that cope with staying at home needs [8]. Nevertheless, stronger research evidence is required to determine the clinical- and cost-effectiveness of these AT devices for types and degrees of disabilities and elderly conditions.

Successful adoption of AT depends on other considerations including individual-specific factors. For instance, sensor-based neuroprostheses are already available commercially, but their high cost not only of their production but also customization is a significant barrier to adoption. In addition, this type of AT may not be suitable for children due to their complex control and the necessity of frequent device changes to meet the rapid growth needs of children. Neuroprostheses still require further research and development efforts to address these issues [9].

The main goal of the announced special collection on AT (https://www.biomedcentral.com/collections/TRATPD) is to bring under focus contributions from prominent researchers, industry professionals, government regulators, care givers, and other stakeholders in the field of AT. By analyzing these contributions and comprehending their position as related to different phases of development and commercialization, it would be possible to gain insight into the obstacles and factors that affect this process. This understanding could potentially aid all stakeholders in devising plans to help create and successfully commercialize AT devices that are of practical value and accessible globally at an affordable price. Such collective efforts can be a significant driver towards better societal integration of PWDs and greater independence for the elderly.

## Data Availability

Not applicable.

## Abbreviations

AT: Assistive Technology
BCI: Brain Computer Interface
GPS: Global Position System
IoMT: Internet of Medical Things
PWDs: People With Disabilities
